# Age-Related Physiologic Thinning Rate of the Retinal Nerve Fiber Layer in Different Levels of Myopia

**DOI:** 10.1155/2020/1873581

**Published:** 2020-01-20

**Authors:** Daun Jeong, Kyung Rim Sung, Youn Hye Jo, Sung-cheol Yun

**Affiliations:** ^1^Department of Ophthalmology, College of Medicine, University of Ulsan, Asan Medical Center, Seoul, Republic of Korea; ^2^Department of Clinical Epidemiology and Biostatistics, Department of Ophthalmology, College of Medicine, University of Ulsan, Asan Medical Center, Seoul, Republic of Korea

## Abstract

**Purpose:**

To investigate the effect of refractive error on the physiologic thinning rate of the retinal nerve fiber layer (RNFL) in healthy eyes. *Materials and Methods*. This study analyzed 223 eyes of 141 healthy subjects followed for more than 5 years and underwent at least five serial spectral domain optical coherence tomography (SD-OCT) examinations. Longitudinal RNFL measurements were analyzed by linear mixed models incorporating follow-up duration, baseline RNFL thickness, spherical equivalent (SE), age, intraocular pressure, and visual field mean deviation. Thinning rates were classified according to SE into three groups: nonmyopic (NM; >0 D), mild-to-moderately myopic (MM; >–6 D and ≤0 D), and highly myopic (HM; ≤–6 D).

**Results:**

The overall slopes of change in RNFL thickness over time in the NM, MM, and HM groups were −0.305 ± 0.128, −0.294 ± 0.068, and −0.208 ± 0.097 *μ*m/yr, respectively. Slopes of RNFL thickness changes in these groups were −0.514 ± 0.248, −0.520 ± 0.133, and −0.528 ± 0.188 *μ*m/yr, respectively. Slopes of RNFL thickness changes in these groups were −0.514 ± 0.248, −0.520 ± 0.133, and −0.528 ± 0.188 *μ*m/yr, respectively. Slopes of RNFL thickness changes in these groups were −0.514 ± 0.248, −0.520 ± 0.133, and −0.528 ± 0.188 *μ*m/yr, respectively. Slopes of RNFL thickness changes in these groups were −0.514 ± 0.248, −0.520 ± 0.133, and −0.528 ± 0.188 *μ*m/yr, respectively. Slopes of RNFL thickness changes in these groups were −0.514 ± 0.248, −0.520 ± 0.133, and −0.528 ± 0.188

**Conclusions:**

Refractive error did not affect the physiologic thinning rate of RNFL when assessed by SD OCT.

## 1. Introduction

Glaucoma is a progressive optic neuropathy, which can lead to blindness in the absence of timely treatment. Although optimal treatment requires a determination of glaucomatous deterioration, this determination is hindered by the concomitant occurrence of age-associated physiologic deterioration. Normal subjects have shown age-associated progressive thinning of their retinal nerve fiber layer (RNFL) and reductions in visual field (VF) sensitivity [1–7]. Thus, in defining glauomatous progression, it is important to consider this physiologic worsening rate, such that progressive changes exceeding the physiologic thinning rate can be regarded as pathologic glaucomatous deterioration.

Myopia is a risk factor for primary open angle glaucoma (POAG), and a high proportion of patients with POAG in clinic is myopic [8–11]. Hence, understanding the characteristics of myopic eyes is essential for the treatment of myopic POAG patients. Although RNFL is thinner in myopic than in nonmyopic eyes [12, 13], it remains unclear, whether the physiologic thinning rate of the RNFL is faster in myopic than in nonmyopic eyes. If myopic eyes have a faster physiologic thinning rate, this rate should be considered when determining glaucomatous progression in myopic POAG patients. Therefore, the current study assessed the longitudinal RFNL thinning rates in normal healthy eyes as a function of refractive error, as determined by spectral domain optical coherence tomography (SD-OCT).

## 2. Methods

### 2.1. Subjects

Subjects at the Asan Medical Center who underwent ocular health screening and were followed up by Cirrus HD-OCT (Carl Zeiss Meditec Inc., Dublin, CA, USA) at least five times for more than 5 years between January 2009 and March 2018 were included in this study. The medical records of these subjects were reviewed retrospectively by a single glaucoma specialist (DJ). Initial testing included a comprehensive ophthalmological examination, including a review of medical history, measurement of best-corrected visual acuity (BCVA), slit lamp biomicroscopy, and multiple measurements of intraocular pressure (IOP) by Goldmann applanation tonometry, gonioscopy, dilated funduscopic examination, stereoscopic optic disc photography, red-free RNFL photography, standard automated perimetry (SITA Standard 24-2; Carl Zeiss Meditec Inc.), and Cirrus HD-OCT.

All eyes had a BCVA of ≥20/30 with a normal anterior chamber and normal retinae on slit lamp examination and fundus photography. Subjects with an abnormal appearing optic disc or an RNFL defect were excluded after careful review of the optic disc/RNFL photography results by two glaucoma specialists (KRS and DJ). Also excluded were subjects who were eligible at initial testing but who later developed a VF or RNFL defect or a glaucomatous optic disc during the follow-up period. Eyes with retinal pathology, such as an epiretinal membrane or diabetic retinopathy, were also excluded. Eyes with a history of refractive surgery, intraocular surgery, or retinal laser therapy at initial work up, as well as those that underwent these procedures any time during the follow-up period were also excluded. Hence, eyes that were aphakic or pseudophakic at baseline examination were also excluded.

Eyes were divided into the three groups based on the level of baseline spherical equivalent (SE): nonmyopic (NM, >0 D), mild-to-moderate myopic (MM, >–6 D< and ≤0 D), and highly myopic (HM; ≤–6 D). The study protocol was approved by the Institutional Review Board of the Asan Medical Center, and the study was performed in accordance with principles of the Declaration of Helsinki.

### 2.2. SD-OCT Imaging

SD-OCT images were obtained as described using a Cirrus HD-OCT system, which is calibrated regularly by a technician employed by the manufacturer [14–16]. RNFL thicknesses were obtained using the optic disc cube mode. All included eyes exhibited a centered optic disc and were well focused with even and adequate illumination, did not move within the measurement circle and had a signal strength of at least seven. RNFL thickness was measured in four quadrants (temporal, superior, nasal, and inferior). For inclusion in analysis, at least five acceptable SD-OCT images of each eye, taken at separate visits, were required.

### 2.3. Statistical Analysis

Continuous variables are reported as mean ± standard deviation and categorical variables as number (frequency). The overall progression rates of RNFL thickness were determined from serial measurements of each parameter using a linear mixed effect model (LMM). Models were fitted with fixed coefficients (fixed effects) of participant baseline RNFL thickness, SE, age, gender, IOP measurements, visual field mean deviation (VF MD), time (i.e., follow-up duration), and the interactions of SE, age, gender, IOP, VF MD, and follow-up duration with random intercepts. Residual diagnostic plots were used to detect features of concern in the model. Exploratory analyses of the residuals suggested that the chosen models were appropriate. The adjusted slopes of the average RNFL thicknesses and of the RNFL thicknesses in each quadrant were compared among the NM, MM, and HM groups using a testing interaction term in the LMM. All reported *P* values were two-sided, and a value of *P* < 0.05 was considered statistically significant. All statistical analyses were performed using SAS software, version 9.2 (SAS Institute, Inc, Cary, NC).

## 3. Results

Over a mean follow-up period of approximately 6 years, 10 eyes of nine subjects who were eligible for inclusion at baseline examination developed glaucomatous optic disc changes and were therefore excluded. Thus, 1407 images of 223 eyes of 141 healthy subjects were analyzed. All 141 subjects were East Asians (Koreans), including 74 (52.5%) men and 67 (47.5%) women. Of the 141 subjects, 11 (7.8%) had diabetes mellitus, 30 (21%), systemic hypertension, and 9 (6%) had a family history of glaucoma. Of the 223 eyes, 37 (16.6%), 124 (55.6%), and 62 (27.8%) were classified by SE into the NM, MM, and HM groups, making 83.4% of all eyes myopic. Characteristics of the study participants are summarized in Table 1. Each eye underwent an average of 6.31 OCT examinations, and the mean duration of follow-up was 5.8 ± 0.7 years. At baseline, the mean SE was −4.0 ± 3.6 D and the mean IOP was 16 ± 2.9 mmHg, with a mean follow-up IOP of 15 ± 2.3 mmHg. Mean VF MD at baseline was −0.79 ± 1.24 dB, and mean RNFL thickness was 85.8 ± 9.8 *μ*m.  Table 2 shows the baseline demographic and clinical characteristics of the three subgroups based on SE. Age at baseline was higher in the NM than in the MM and HM groups.

The change of RNFL thickness over time in all 223 eyes was estimated using an LMM (Table 3), which showed a significant interaction between thinning rate and time. Overall slope over time was -0.389 *μ*m/yr (*P* < 0.0001) in men and −0.164 *μ*m/yr in women, a difference of 0.225 *μ*m/yr that was statistically significant (*P*=0.006). The slopes of RNFL thickness in the superior, temporal, and inferior quadrants, but not in the nasal quadrant, showed significant interactions with time. The changes in slope over time were −0.524 *μ*m/yr in the superior sector and −0.778 *μ*m/yr in the inferior sector, with none of the analyzed variables influencing these slopes. In the temporal sector, the RNFL thickness was 0.034 *μ*m/yr less reduced as myopia increased by one diopter, resulting in a rate of −0.316 (−0.034) = −0.282 *μ*m/yr (*P*=0.040), with the rate of reduction being 0.345 *μ*m/year greater in men than in women (*P*=0.006).

We compared the rate of changes in RNFL thickness in the three SE groups after adjusting for between-eye interactions within each subject, sex, age, SE, IOP, mean follow-up IOP, baseline RNFL thickness, VF MD, and laterality (Table 4). The overall slopes of change in RNFL thickness over time in the NM, MM, and HM groups were −0.305 ± 0.128, −0.294 ± 0.068, and −0.208 ± 0.097 *μ*m/yr, respectively. The slopes of RNFL thickness changes in these groups were −0.514 ± 0.248, −0.520 ± 0.133, and −0.528 ± 0.188 *μ*m/yr, respectively, in the superior quadrant; −0.084 ± 0.145, 0.107 ± 0.082, and −0.161 ± 0.112 *μ*m/yr, respectively, in the temporal quadrant; −0.807 ± 0.242, −0.794 ± 0.130, and −0.727 ± 0.183 *μ*m/yr, respectively, in the inferior quadrant; and 0.160 ± 0.157, 0.118 ± 0.084, and 0.429 ± 0.119 *μ*m/yr, respectively in the nasal quadrant. Overall and in each quadrant, there was no significant difference in the rate of RNFL thickness change among the three SE-based groups.

## 4. Discussion

This longitudinal analysis of 223 healthy eyes for approximately 6 years showed that average RNFL thickness decreased at rates of 0.208–0.305 *μ*m/yr after adjustment for covariates. In comparison, cross-sectional studies that included subjects of various ages found that average RNFL thickness decreased at rates of 0.16–0.26 *μ*m/yr, slightly less than in our study [1, 3, 4, 17]. However, a longitudinal study that investigated RNFL changes in normal eyes of Hong Kong Chinese, found that the rate of average RNFL thickness change was −0.33 *μ*m/yr [2], and a study similar to ours reported a rate of −0.54 *μ*m/yr [6], Thus, in general, thinning rates have been higher in longitudinal than in cross-sectional studies. Cross-sectional studies, however, cannot determine the true rate of change over time in the same participants; rather, they compare slopes of RNFL thickness in various age groups. Thus, the actual age-associated rate of reduction of RNFL thickness in normal eyes is approximately 0.3 *μ*m/yr.

RNFLs are thinner in myopic than in nonmyopic eyes, with thickness associated with the degree of myopia [13, 18, 19]. Although baseline RNFL in our study subjects was thinnest in the HM group, refractive error was not associated with the overall thinning rate of RNFL during follow-up. Moreover, a comparison of the adjusted slopes of RNFL change showed no significant differences among the three SE-based groups. The reason for the inverse association between RNFL thickness and degree of myopia is unclear. However, myopic axial elongation may subject retinal nerve fibers to tensile stress, which may eventually damage the retinal nerve fibers and reduce RNFL thickness. Because this myopic axial elongation usually occurs during childhood or early adolescence, the normal thinning rate may not have been affected by myopic axial elongation in our participants, whose mean baseline age was 40 years. In addition, myopic glaucomatous eyes have thinner RNFLs, with myopia being a risk factor for glaucoma development [20, 21]. However, myopia was not associated with faster progression of glaucoma in these studies, although previous studies have yielded inconsistent results [22]. Myopic axial elongation may damage the RNFL, thereby affecting the development of glaucoma in some young patients [8, 20, 23, 24]. However, because myopic elongation stops at adolescence, subjects with myopic glaucoma may not show rapid deterioration [25]. Similarly, our results may suggest that although the RFNL tended to be thinner in healthy myopic than in healthy nonmyopic eyes and that RNFL tended to be thinner as the degree of myopia increases, these myopic eyes do not show a more rapid age-related rate of RNFL thinning because myopic elongation stops or slows when one becomes an adult.

Examination of the four quadrants of the eyes included in this study showed that thinning rate was fastest in the inferior quadrant (−0.778 *μ*m/yr), followed by the superior quadrant (−0.524 *μ*m/yr). These quadrants were previously reported to have faster thinning rates than the nasal and temporal quadrants [2]. The faster rate of RNFL thinning in the superior and inferior quadrants may be due to the structural weakness of these areas in the optic nerve head lamina cribrosa. The superior and inferior regions of the lamina cribrosa have been reported weaker, making these regions more vulnerable to glaucomatous damage [22, 26]. This relative weakness of support tissue can also affect physiologic thinning in healthy eyes.

In contrast to findings in the inferior and superior quadrants, the nasal quadrant showed no significant changes over time in RNFL thickness. Similarly, thickening of the RNFL in this quadrant by +0.308 *μ*m/yr has been reported [2], further suggesting that the nasal sector did not experience age-related loss in RNFL thickness. This may have been due to the proportion of nonneuronal tissue, such as glial tissue, in the RNFL, which has been reported to increase with age [27, 28]. OCT assesses the thickness between the internal limiting membrane and the ganglion cell layer in the retina; thus, OCT cannot measure the RNFL separately from other layers. Axonal fibers in the RNFL decrease with age, indicating an inverse relationship between thickness and the proportion of nonneuronal tissue. Thus, changes in RNFL, as measured by OCT, result from a combination of a decreased width of neuronal tissue and an increased width of nonneuronal tissue. This would apply not only to the nasal quadrant but also to all quadrants. However, consistent results showing that RNFL thickness in the nasal sector increases or remains stable suggest an effect of nonneuronal tissue and its possible increase over time.

In contrast to our results, RNFL thinning rate has been reported to be faster in highly myopic than in nonmyopic eyes in subjects aged 40–59 years who were followed-up for more than 3 years [29]. Because our subjects were relatively younger (mean age, 40 ± 12 years), direct comparison between these studies may be inappropriate. Furthermore, the previous study also reported that RNFL thinning rates were similar in highly myopic and nonmyopic eyes of younger subjects, in agreement with our results [29]. Furthermore, the shorter follow-up period in the previous study (3 years) than in ours (6 years) may have resulted in different outcomes. Highly myopic eyes with injured and thinner RNFL in younger subjects due to a sudden increase in axial length may experience a faster deterioration of the RNFL as the subject becomes 40–59 years old. Further studies are needed, however, to test this hypothesis. Our participants showed faster thinning in men compared with women. This may be due to thinner baseline RNFL in men than in women (83.4 vs. 87.7 *μ*m).

To our knowledge, this is the first study to compare RNFL changes as a function of refractive error in healthy eyes for a relatively long period of time. Although myopic eyes are more vulnerable to glaucomatous changes [30–33], we found that, after controlling for confounding variables, refractive error had no impact on RNFL thickness change in normal eyes.

This study had several limitations. Our population consisted of normal subjects; nonetheless, it was a retrospective analysis of participants who had been followed-up annually during routine check-ups. All eyes with abnormally appearing optic discs were excluded. However, our participants may not represent the normal population. Moreover, all of the subjects in our study were East Asian, an ethnic population with a high prevalence of myopia, indicating the need to assess the association between myopia and the rate of change in RNFL thickness in other ethnic groups [34, 35]. In addition, we originally intended to investigate the relationship between axial length and age-related RNFL loss. However, as axial length was not measured in some participants, we assessed SE rather than axial length.

In summary, this study assessed the rate of age-related RNFL loss in normal, nonglaucomatous eyes. Refractive error did not affect the physiologic thinning of the RNFL, as assessed by SD OCT.

## Figures and Tables

**Table 1:** Demographics and optical coherence tomography circumpapillary retinal nerve fiber layer (RNFL) and macular measurements.

Subjects	223 eyes from 141 individuals
Age (yrs)	40 ± 12
Gender (male: female)	111 : 112
Mean f/u frequency	6.31 ± 1.22
Mean f/u period (yrs)	5.8 ± 0.71
S.E (D)	−4.0 ± 3.6
Baseline IOP (mmHg)	16 ± 2.9
F/u mean IOP (mmHg)	15 ± 2.3
Baseline MD (dB)	−0.79 ± 1.24
avr. RNFL thickness (*μ*m)	85.8 ± 9.81
	Of 141 individuals
DM (%)	7.8 (*n* = 11)
HTN (%)	21 (*n* = 30)
Family history of glaucoma (%)	6 (*n* = 9)

S.E: sepherical equivalent; IOP: intraocular pressure; f/u: follow-up; RNFL: retinal nerve fiber layer; GCIPL: ganaglion cell-inner plexiform layer.

**Table 2:** Clinical characteristics of three subgroups based on SE.

	NMG (A), *n* = 37	MMG (B), *n* = 124	HMG (B), *n* = 62	*P* value (A vs. B, A vs. C, B vs. C)		
Age (yrs)	50 ± 13	39 ± 11	36 ± 12	<0.001	<0.001	0.133
Gender (male:female)	12 : 25	70 : 54	29 : 33	0.354^§^		
S.E (D)	0.69 ± 0.79	−2.96 ± 1.69	−8.7 ± 1.77	<0.001	<0.001	<0.001^*∗*^
Baseline IOP (mmHg)	15 ± 3.7	16 ± 3.0	16 ± 2.2	1.0	1.0	1.0
Follow-up mean IOP (mmHg)	14 ± 2.2	15 ± 2.5	15 ± 2.1	0.373	0.207	1
Baseline MD (dB)	−0.47 ± 1.3	−0.74 ± 1.2	−1.1 ± 1.3	0.733	0.059	0.253
Global RNFL thickness (*μ*m)	90.2 ± 9.5	86.9 ± 10.3	82.1 ± 7.6	0.108	<0.001	0.009
RNFL thickness, superior quad. (*μ*m)	113.1 ± 17.1	107.4 ± 17.4	100.0 ± 13.8	0.207	0.001	0.017
RNFL thickness, temporal quad. (*μ*m)	67.4 ± 8.3	67.5 ± 12.5	71.8 ± 14.0	1.0	0.281	0.089
RNFL thickness, inferior quad. (*μ*m)	118.2 ± 16.7	110.0 ± 17.6	99.0 ± 13.5	0.02	<0.001	<0.001
RNFL thickness, nasal quad. (*μ*m)	62.2 ± 8.4	62.9 ± 9.7	57.5 ± 9.0	1.0	0.056	0.001

SE: spherical equivalent, D = diopters; IOP = intraocular pressure; MD = mean deviation; RNFL = retinal nerve fiber layer, quad. = quadrant. Data are presented as mean values ± standard deviation. Comparison among three groups was performed by analysis of variance with the Bonferroni method. ^§^*P* value with the linear-by-linear association test. ^*∗*^*P* values with the Dunnett T3 method.

**Table 3:** Retinal nerve fiber layer thinning rates (*µ*m/year) calculated by linear mixed effect model.

	Average of all sectors			Superior sector			Temporal sector			Inferior sector			Nasal sector		
	Beta	SE	*P* value	Beta	SE	*P* value	Beta	SE	*P* value	Beta	SE	*P* value	Beta	SE	*P* value
Intercept	102.170	5.404	<.0001	133.850	9.958	<.0001	88.397	6.739	<.0001	140.350	9.829	<.0001	42.930	5.590	<.0001
age (years)	−0.206	0.069	0.003	−0.417	0.111	<.0001	−0.250	0.088	0.005	−0.327	0.123	0.008	0.139	0.068	0.041
Female	4.279	1.528	0.005	4.213	2.394	0.079	5.799	2.024	0.004	9.122	2.683	0.001	−0.834	1.470	0.571
Right eye	0.371	0.252	0.142	−3.260	0.485	<.0001	3.007	0.311	<.0001	−0.206	0.483	0.670	1.988	0.293	<.0001
Spherical equivalent (D)	0.287	0.176	0.103	1.471	0.299	<.0001	−1.287	0.220	<.0001	0.884	0.320	0.006	0.199	0.182	0.275
Baseline IOP (mmHg)	−0.081	0.223	0.716	−0.184	0.410	0.654	−0.953	0.275	0.001	0.212	0.420	0.614	0.573	0.249	0.021
Mean F/U IOP (mmHg)	−0.512	0.350	0.144	−0.443	0.622	0.477	−0.333	0.433	0.443	−1.540	0.650	0.018	0.322	0.379	0.396
VF MD (dB)	0.504	0.196	0.010	1.054	0.370	0.005	−0.336	0.241	0.164	1.426	0.373	<.0001	0.286	0.267	0.285

Time	−0.389	0.074	<.0001	−0.524	0.100	<.0001	−0.316	0.110	0.005	−0.778	0.099	<.0001	0.121	0.073	0.100
Female	0.225	0.103	0.030	—	—	—	0.345	0.124	0.006	—	—	—	—	—	—
Age (years)	—	—	—	—	—	—	—	—	—	—	—	—	—	—	—
Spherical equivalent (D)	—	—	—	—	—	—	−0.034	0.017	0.040	—	—	—	—	—	—
Mean F/U IOP (mmHg)	—	—	—	—	—	—	—	—	—	—	—	—	—	—	—
VF MD (dB)	—	—	—	—	—	—	—	—	—	—	—	—	−0.116	0.048	0.017

SE: standard error; D: diopter; IOP: intraocular pressure; MD: mean deviation.

**Table 4:** Rates of change in RNFL thickness of the three myopia groups based on spherical equivalent.

	NM		MM		HM		*P* value
	Slope	SE	Slope	SE	Slope	SE	
Superior	−0.514	0.248	−0.520	0.133	−0.528	0.188	0.999
Temporal	−0.084	0.145	0.107	0.082	−0.161	0.112	0.111
Inferior	−0.807	0.242	−0.794	0.130	−0.727	0.183	0.947
Nasal	0.160	0.157	0.118	0.084	0.429	0.119	0.094
Average	−0.305	0.128	−0.294	0.068	−0.208	0.097	0.738

NM: nonmyopic; MM: mild-to-moderately myopic; HMG: highly myopic.

## Data Availability

The data are available on reasonable request from the corresponding author.
